# In Silico and In Vitro Analysis of lncRNA XIST Reveals a Panel of Possible Lung Cancer Regulators and a Five-Gene Diagnostic Signature

**DOI:** 10.3390/cancers12123499

**Published:** 2020-11-24

**Authors:** Periklis Katopodis, Qiduo Dong, Heerni Halai, Cristian I. Fratila, Andreas Polychronis, Vladimir Anikin, Cristina Sisu, Emmanouil Karteris

**Affiliations:** 1 Biosciences, College of Health, Medicine and Life Sciences, Brunel University London, Uxbridge UB8 3PH, UK; periklis.katopodis@brunel.ac.uk (P.K.); 1706896@brunel.ac.uk (Q.D.); 1710660@brunel.ac.uk (H.H.); 1607992@brunel.ac.uk (C.I.F.); v.anikin@rbht.nhs.uk (V.A.); cristina.sisu@brunel.ac.uk (C.S.); 2 Division of Thoracic Surgery, The Royal Brompton & Harefield NHS Foundation Trust, Harefield Hospital, London UB9 6JH, UK; 3 Mount Vernon Cancer Centre, Northwood HA6 2RN, UK; andreas.polychronis@nhs.net; 4 Department of Oncology and Reconstructive Surgery, Sechenov First Moscow State Medical University, 119146 Moscow, Russia

**Keywords:** XIST, x-inactivation center, NSCLC, lncRNA, TCGA, GTEX, RNA-seq, lung cancer, bioinformatics

## Abstract

**Simple Summary:**

Long non-coding RNAs (lncRNA) have been associated with a number of diseases including cancer. A well-studied lncRNA called XIST (X-inactive specific transcript) acts as a major effector of the X-inactivation process. It is expressed on the inactive X chromosome providing a dosage equivalence between males and females. Recently XIST has been implicated in the development of lung cancer. Using a bioinformatics approach, we demonstrate the XIST is over-expressed in female patients compared to males. When XIST gene was silenced in two different cell lines (of male and female origin), a number of genes were differentially expressed; playing a role in signal transduction pathways, energy balance and metabolism, thus providing a better insight of the role of this lncRNA in cancer. Finally, we showed that expression of XIST with another 4 genes provided a strong diagnostic potential to discriminate lung cancer from healthy controls.

**Abstract:**

Long non-coding RNAs (lncRNAs) perform a wide functional repertoire of roles in cell biology, ranging from RNA editing to gene regulation, as well as tumour genesis and tumour progression. The lncRNA X-inactive specific transcript (XIST) is involved in the aetiopathogenesis of non-small cell lung cancer (NSCLC). However, its role at the molecular level is not fully elucidated. The expression of XIST and co-regulated genes TSIX, hnRNPu, Bcl-2, and BRCA1 analyses in lung cancer (LC) and controls were performed in silico. Differentially expressed genes (DEGs) were determined using RNA-seq in H1975 and A549 NSCLC cell lines following siRNA for XIST. XIST exhibited sexual dimorphism, being up-regulated in females compared to males in both control and LC patient cohorts. RNA-seq revealed 944 and 751 DEGs for A549 and H1975 cell lines, respectively. These DEGs are involved in signal transduction, cell communication, energy pathways, and nucleic acid metabolism. XIST expression associated with TSIX, hnRNPu, Bcl-2, and BRCA1 provided a strong collective feature to discriminate between controls and LC, implying a diagnostic potential. There is a much more complex role for XIST in lung cancer. Further studies should concentrate on sex-specific changes and investigate the signalling pathways of the DEGs following silencing of this lncRNA.

## 1. Introduction

The large-scale genome sequencing studies of the last decade have documented the pervasive transcription of almost 90% of the human genome [1], with 98% of the transcriptome consisting of long non-coding RNAs (lncRNAs) [2]. However, despite these efforts, our current understanding of lncRNAs’ complex biology is still limited. Long non-coding RNAs are a class of RNA molecules that do not encode proteins and range in length from 200 nucleotides to 100 kilobases [3]. Previous analyses have pointed to a wide range of functions for lncRNAs in developmental and cellular processes, including gene expression, chromatin remodeling and modification, splicing, editing, translation and degradation of the RNA, and gene silencing with endogenous small interfering RNA (siRNA) [4,5,6].

LncRNAs were found to be expressed in a variety of diseases including cancer suggesting potential roles as biomarkers or even therapeutic targets. Studies have identified key lncRNAs as regulators of oncogenes and tumour suppressors such as PTEN and KRAS [7,8,9]. Other lncRNAs were shown to be important for genomic imprinting, and for regulating epigenetic procedures. This is the case for lncRNA X-inactive specific transcript (XIST) that inactivates one of the two X chromosomes in females [10,11,12].

**Figure 1:**
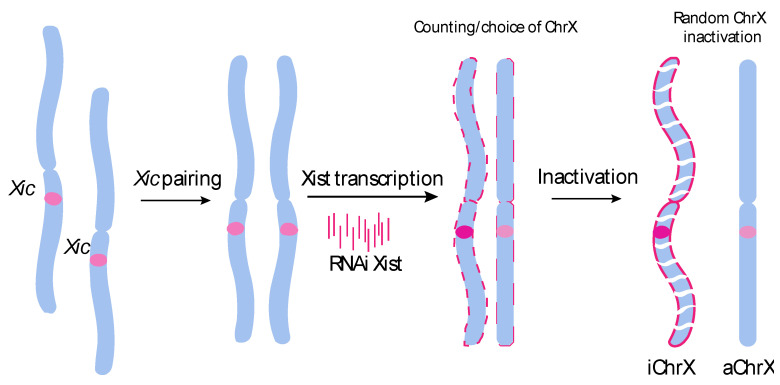
Process of X chromosome inactivation (Xi). XIST is triggering an X chromosome inactivation while the other X chromosome remains active. XIST is spreading along one of the X chromosomes (coating) and its binding triggers the chromatin inactivation. Xic: X-inactivation center, iChrX: inactive Xchr, aChrX: active Xchr [14].

In literature, XIST is described interchangeably as either a lncRNA or a pseudogene and acts as a major effector in the X chromosome inactivation process. It is expressed only on the inactive X chromosome providing a dosage equivalence between males and females (reviewed in [10,13]). XIST was the first non-coding gene identified in the X inactivation center (XIC) region [14]. The expression of this lncRNA is the essential step for the initiation of the X inactivation. XIST is transcribed, spliced, and polyadenylated resulting in an mRNA. However, no protein products have ever been observed. XIST is coating and inactivates one of the X chromosomes (Figure 1). However, the lack of XIST activity leads to the failed inactivation and duplication of the gene on the second X chromosome, resulting in its activation [14,15].

Another key lncRNA that mediates the X chromosome inactivation is TSIX. TSIX acts as a XIST repressor. TSIX is the antisense RNA of XIST and their differential expression patterns define the activation or inactivation of the X chromosome. Apart from the XIST-TSIX mechanism, XIST RNA and the nuclear matrix protein heterogeneous nuclear ribonucleoprotein U (hnRNPu) interact and, upon the depletion of hnRNPu, XIST is detached from the inactive X chromosome (Xi) and diffusely localized into the nucleoplasm [10,15].

Recent studies have shown that *XIST* has an aberrant expression pattern in breast cancer [16], cervical squamous cell carcinoma [17], colorectal cancer [18], gastric cancer [19], glioma [20], hepatocellular carcinoma [21], nasopharyngeal carcinoma [22], non-small cell lung cancer (NSCLC) [23], pancreatic cancer [24], osteosarcoma [25], and ovarian cancer [26]. Furthermore, XIST was shown to regulate tumour cell migration, proliferation, and invasion, in NSCLC [6,27]. Wang et al. showed that cell lines and patient samples of NSCLC overexpressed *XIST* and shown that *XIST* knockdown inhibits tumour growth in vivo. Moreover, XIST exhibits oncogenic properties by regulating the miR-449a and B-cell lymphoma 2 (Bcl-2) gene in NSCLC [5]. The same study has shown that BRCA1 also influences the concentration of XIST on the Xi. Specifically, RNAi of BRCA1 decreases the concentration of XIST on Xi, and the reduction of BRCA1 by Cre-mediated excision also decreases XIST concentration on Xi [28]. Collectively these data indicate that XIST could be an important novel biomarker for the detection of NSCLC [23,29]. We hypothesize that there is a higher order of complexity in the regulation of XIST and its impact in multiple signaling pathways. In this study, we investigate changes in the transcriptional landscape of cell lines from male and female patients with NSCLC where XIST was downregulated and studied the expression and correlation of associated genes in lung cancer.

## 2. Results

### 2.1. Expression Level of XIST, TSIX, hnRNPu, Bcl-2, and BRCA1 in NSCLC

Leveraging the available expression data from TCGA and GTEX, as tumour and respectively normal control lung samples (referring as ‘Lung’ in figures), we have investigated differential expression patterns of *XIST*, *TSIX*, *hnRNPu*, *Bcl-2*, and *BRAC1* in lung adenocarcinoma (LUAD) and lung squamous carcinoma (LUSC) (Supplementary Figure S1).

**Figure 2:**
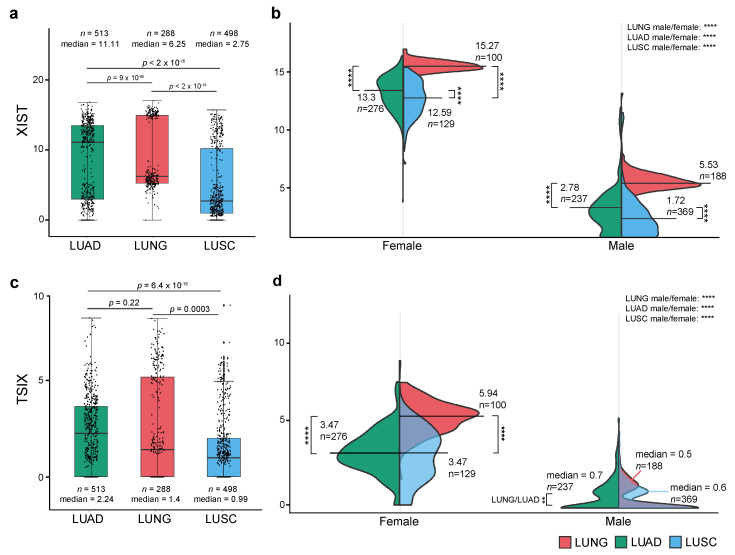
*XIST* and *TSIX* expression. (**a**) Boxplots of the overall *XIST* expression in LUAD, LUSC and normal lung (LUNG), (**b**) Violin plots of the gender-specific expression of XIST in LUAD, LUSC and LUNG, (**c**) Boxplots of the overall *TSIX* expression in LUAD, LUSC, and LUNG (**d**) Violin plots of the gender-specific expression of *TSIX* in LUAD, LUSC, and normal lung. All expression data is presented in units of log2(norm_count + 1). Median expression and total sample size (count, n) are shown. *p*-values were calculated the Wilcoxon test in R, statistically, significant represented as ****: *p* ≤ 0.0001, non-significant results are not labelled in the graph.

Overall, similar to previously published results where XIST is upregulated in various cancers [6,30], we found a similar trend, in LUAD compared to normal lung cohort (LUNG), whereas in LUSC a downregulation was observed (Figure 2a). However, given the involvement of XIST in X chromosome inactivation, we dissected these data further by measuring the expression of *XIST* in males and females. Here, a different picture emerges, suggesting a gender-specific downregulation in tumour samples compared to control Figure 2b. For *TSIX*, a modest overall upregulation in LUAD was observed, whereas in LUSC a significant downregulation when compared to normal lung (LUNG) was noted (Figure 2c). Further gender stratification showed the emergence of a similar distribution as noted earlier for *XIST* (Figure 2d).

**Figure 3:**
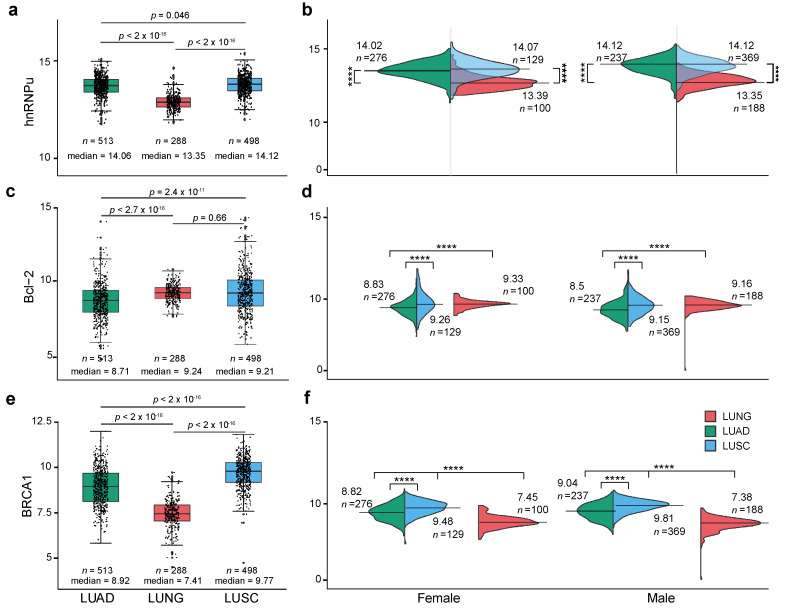
Gene expression patterns for (**a**,**b**) *hnRNPu*, (**c**,**d**) *Bcl-2* and (**e**,**f**) *BRCA1* in units of log_2_(norm_count + 1) for normal lung, lung adenocarcinoma (LUAD) and lung squamous cell carcinoma (LUSC). Median expression and sample size were labelled. Median expression and total sample size (count, n) are shown. *p*-values were calculated the Wilcoxon test in R, statistically, significant represented as ****: *p* ≤ 0.0001, non-significant results are not labelled in the graph.

Next, we investigated the expression patterns of *hnRNPu*, *Bcl-2*, and *BRCA1* in the same cohorts of patients (Figure 3). Overall, we found that the expression of *hnRNPu* and *BRCA1* in LUAD and LUSC is significantly upregulated compared to the normal lung samples in both males and females. On the other hand, *Bcl-2* appears to be downregulated only in LUAD when compared to controls. Moreover, the three genes did not display any gender-specific expression patterns as observed earlier for *XIST* and *TSIX* suggesting that the transcriptional landscape of *hnRNPu*, *Bcl-2*, and *BRCA1* is gender agnostic.

**Figure 4:**
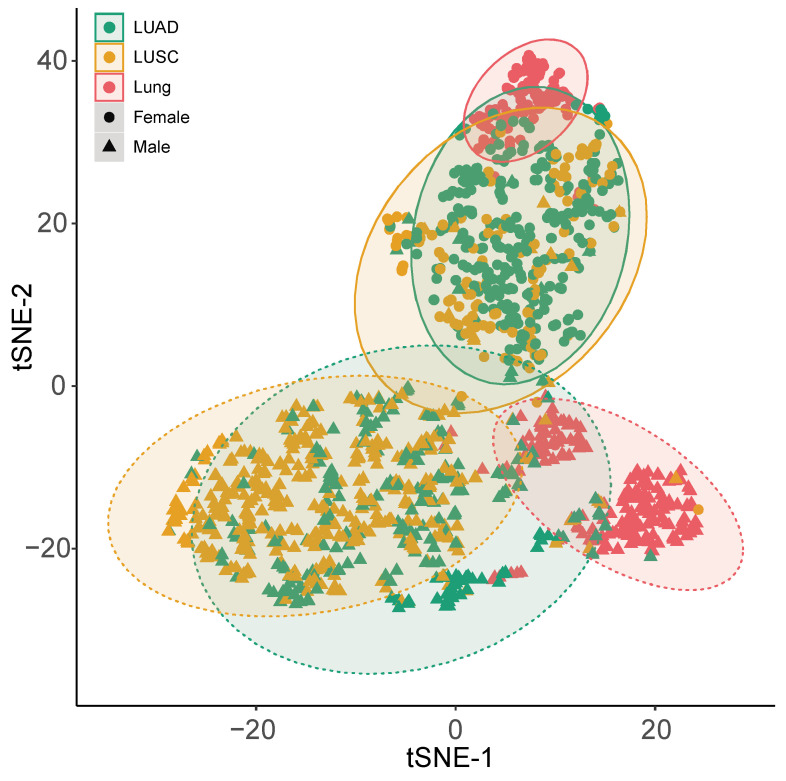
Clustering of tumour and healthy lung samples in a 2D map using t-SNE algorithm based on the normalized expression levels of *XIST, TSIX, hnRNPu*, *Bcl-2*, and *BRCA1*. tSNE-1 and tSNE-2 are the tSNE projection axes.

Following the sample and gender-specific expression analysis, we used T-distributed stochastic neighbor embedding (t-SNE) to evaluate the discriminatory power of the gene expression patterns to differentiate between normal and tumour samples (Figure 4 and Supplementary Figure S2). Visual inspection of the t-SNE plot showed a clear and distinct clustering of male and female samples. Furthermore, by combining the expression information, we were able to discriminate between normal and cancer samples, suggesting a good collective diagnosis biomarker potential for the five genes. Notably, some cancer samples are overlapping healthy samples, suggesting that some healthy individual’s expression pattern of the five genes is similar to the ones observed commonly in tumour samples, hinting at the potential existence of cancer precursors in healthy individuals.

**Figure 5:**
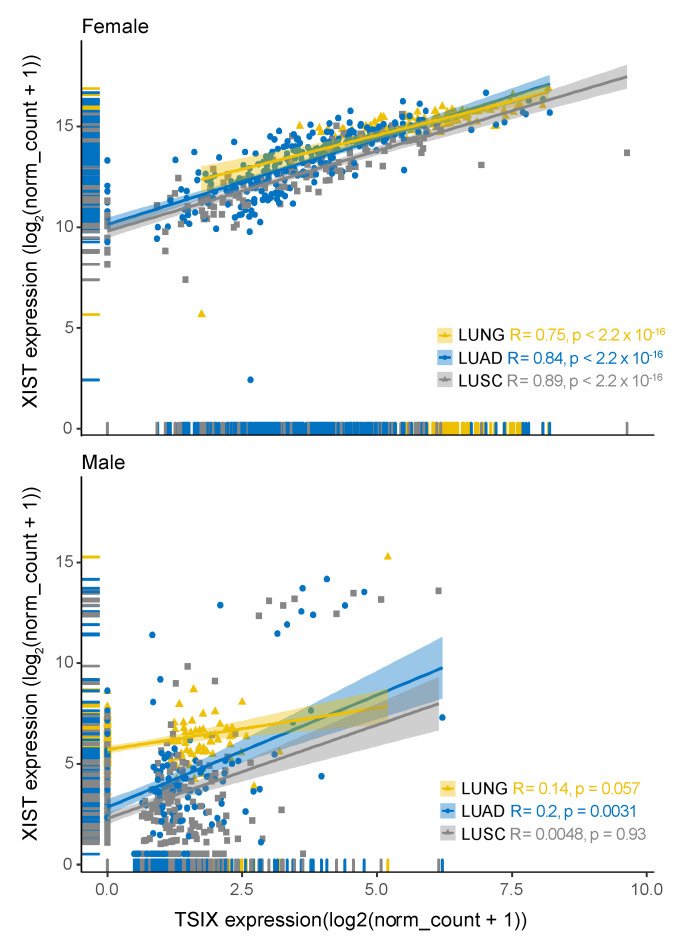
Correlation with regression line for gene expression (in units if log2(norm_count + 1)) for XIST vs. TSIX, Spearman’s rank correlation test was used to generate the correlation coefficient represented as R in the analyses, p represents *p*-value. Healthy lung, LUAD, and LUSC were compared separately in both sexes. Total sample number is 1299, 794 derived from male patients and 505 from female patients. There are 116 samples from male patients with 0 expression in XIST, 461 in TSIX. In female patients derived samples, 17 show zero expression for TSIX gene. All samples with zero expression in both genes were removed from the analysis.

Next, we investigated whether the *XIST* and the four associated genes have correlated expression patterns in NSCLC (Supplementary Figure S3). For this, we used Spearman’s rank correlation tests evaluating male and female samples individually. We found that *XIST* has an independent expression pattern with respect to *hnRNPu*, *Bcl-2*, and *BRCA1*. However, as expected we observed a strong correlation between *XIST* and *TSIX* expressions in samples derived from female patients (R = 0.75 for LUNG cohort, 0.84 for LUAD and 0.89 for LUSC) (Figure 5).

### 2.2. Functional Analysis

**Figure 6:**
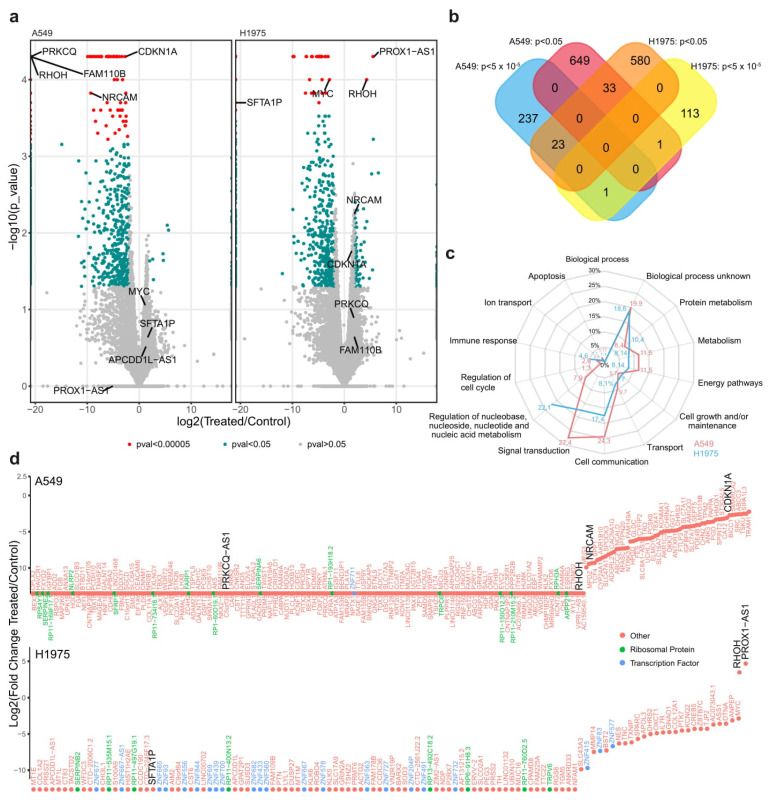
(**a**) Vulcan plots for the A549 and H1975 gene expression. Data shows mostly up-regulation or down-regulation of genes in the siRNA XIST treated samples in comparison to non-treated ones (left of center = down-regulation, right of center = up-regulation), with a handful of genes shown to be very significantly up or down-regulated. (**b**) Venn diagram showing the total genes significantly dysregulated for both cell lines. (**c**) The biological processes where the down-regulated genes are found. Most of the dysregulated genes in both cells lines are playing a significant role in signal transduction (27.4% for the A549), cell-to-cell communication (24.3% for the A549 and 17.4% for the H1975) and regulation of nucleic acid metabolism (22.1% for the H1975 and 7.9% for the A549), (**d**) Ranking of all the highly significantly differentially expressed genes (DEGs) in both cell lines (*p* < 5 × 10^−5^).

Following the sexual dimorphism of XIST, we investigated the change in the genome transcriptional landscape A549 (derived from male patient) and H1975 (derived from female patient) NSCLC cell lines upon silencing *XIST* using siRNA (Figure 6). The differential expressed genes were identified using RNAseq analysis. The results are summarized in the Vulcan plots. Overall, we distinguished three types of genes (Figure 6a). First, there are genes (shown in red) that show a statistically significant (*p*-value < 10^−4^) differential transcription pattern, with a large absolute value of log_2_FoldChange > 2, between the two analyzed conditions. Second, there are genes (seen in green) that show a moderately significant change in expression (*p*-value < 0.05) and an absolute value of log_2_FoldChange > 2. Finally, some genes do not show any significant change in the transcription levels between the analyzed states (shown here in grey).

Overall, 944 genes were significantly dysregulated after the treatment with the siRNA XIST for the A549 cell line. From those genes, the 683 were downregulated at *p* < 0.05, and 261 were highly-significantly down-regulated at *p* < 5 × 10^−5^. In the H1975, 751 genes were significantly dysregulated after the treatment with the siRNA XIST. 536 genes were downregulated at *p* < 0.05 and 113 significantly down-regulated *p* < 5 × 10^−5^ with two genes to present and highly significant upregulation after the transfection. The DEGs are presented as supplementary table. The two cell lines had 34 downregulated genes in common with *p* < 0.05, 24 downregulated genes with *p* < 5 × 10^−5^, and one gene (*RHOH*) that was up-regulated in the H1975 and down-regulated for the A549 (Figure 6b). Subsequent analysis of the differentially expressed genes (DEGs) using FunRich revealed distinct biological processes for the two cell lines, including signal transduction, cell communication, metabolism, and energy pathways (Figure 6c).

**Figure 7:**
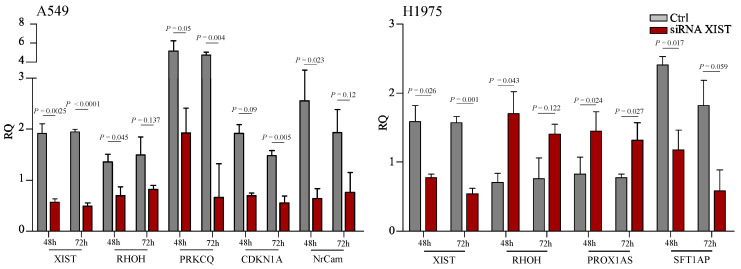
Relative expression of selected targets validating RNAseq data. For A549 cell line, XIST, RHOH, PRKCQ, CDKN1A and NrCam were validated using qRT-PCR. Significant downregulation corroborated the RNA-seq data. Similarly, XIST, RHOH, PROX1AS and SFT1AP were also assessed. Downregulation of XIST and SFT1AP was noted and upregulation of RHOH and PROX1AS following siRNA transfection. These data also corroborate the trends seen in RNA-seq for this cell line.

A summary of the top DEG in the A549 and H1975 cell lines respectively is shown in Figure 6d. In both cell lines, XIST was downregulated in agreement with previous studies that siRNA was used [6,27,31,32]. *PROX1AS1* (antisense RNA) and *RHOH* (a negative regulator of cell growth and survival) were markedly upregulated in H1975 cells in agreement with RNAseq data. We expanded on these observations in A549 cells where *RHOH*, *PRKCQ* (required for the activation of the transcription factors NF-kB and AP-1), *NrCAM* (required for normal responses to cell-cell contacts in the brain and peripheral nervous system), and CDKN1A (involved in p53 mediated inhibition of cellular proliferation to DNA damage) were downregulated in siRNA treated cells in agreement with the general trend observed in RNAseq for this cell line (Figure 7).

## 3. Discussion

In this study, we provide a novel insight into the expression of XIST and key co-expressed (?) genes. Leveraging the available large-scale sequence data from TCGA and GTEX, we demonstrate that *XIST*, *TSIX*, *hnRNPu*, *Bcl-2*, and *BRCA1* are differentially expressed in two different types of lung cancer when compared to controls. Initial observations suggested that XIST was significantly upregulated in LUAD compared to controls. This finding corroborates previous studies where XIST has been shown to be over-expressed in lung cancer and other cancers [23,31,33,34,35,36,37].

However, subsequent stratification by sex revealed that XIST is highly expressed in females and downregulated in LUAD and LUSC when compared to normal controls. Although this might appear as a discrepancy, it is evident that there is a vast range in XIST’s gene expression when all samples are measured independently of gender. Thus, depending on data preparation and processing a potentially different picture can emerge. For example, in all earlier studies, XIST expression was not stratified in male and female lung cancer patients and numbers were considerably lower to the ones assessed in this study. Of note, our detailed analysis revealed the presence of two groups in control and LUAD cohorts (Figure 3a). Apart from issues relating to sex-specific expression, the preparation of RNA and subsequent analyses of data might also be contributing factors for the detection of lncRNAs. It has been recommended -when using sequencing for lncRNAs- that rRNA depletion (instead of polyA selection) should be performed [38]. If RNA-seq is polyA+ enriched, it will bias the analysis. It has also been suggested that 50 million paired reads are needed for tissues, as there are so many and low abundant lncRNAs. Another factor that can potentially induce different results is the method used for the quantification of lncRNAs [39]. Our data provides a deeper insight into the differential expression of *XIST* and highlights the need of standardization of RNA preparation protocols that would ultimately increase the transcriptomic comparability between different RNA-seq datasets, thus enabling a better mapping of lncRNAs [40,41].

Clustering of tumour and healthy lung samples in a 2D map using t-SNE algorithm based on the normalized expression levels of *XIST*, *TSIX*, *hnRNPu*, *Bcl-2*, and *BRCA1*, revealed that collectively these five genes -when assessed together- can have a diagnostic potential in both LUAD and LUSC. This observation warrants further investigation in both tissue and liquid biopsies from NSCLC patients using a much larger cohort especially for the healthy control group. Further, a strong correlation between *XIST* and *TSIX* was seen. *TSIX* is the antisense of *XIST*. It is the complementary sequence to *XIST* that is 40 kilobases long as is transcribed in the opposite direction across the *XIST* gene. Much like *XIST*, *TSIX* only acts on the chromosome it is produced by. The relationship between *TSIX* and *XIST* is inverse meaning that when the expression of *TSIX* is increased, *XIST* expression is reduced therefore it blocks inactivation in the cis or same X chromosome. When *TSIX* expression is reduced, *XIST* expression is increased and causes the inactivation of the X chromosome [4].

Silencing *XIST* in vitro resulted in 944 DEGs for A549 and 751 DEGs for H1975 cell lines respectively. A common trend for both cell lines was that the majority of DEGs were downregulated compared to controls, providing further evidence for a critical role of XIST in cell proliferation as it has been shown in previous studies [6].

For example, in A549 cells, genes like protein kinase C theta (*PRKCQ*/*PKC-θ*), cyclin-dependent kinase inhibitor 1A (*CDKN1A*/*p21*), neuro-glia-related cell-adhesion molecule (*NrCAM*) and ras homolog family member H (*RHOH*) were amongst the most downregulated genes. PKC-θ enhances anchorage-independent survival, growth-factor-independent proliferation, and migration and its down-regulation enhances anoikis [42]. In addition PKC-θ regulates cell cycle checkpoint pathways in lung cancer [43]. Cyclin-dependent kinase inhibitor 1A (*p21*) can exert multiple roles including progression of the cell cycle, DNA repair, apoptosis, and can also function as an oncogene which promoting tumour growth by inhibiting apoptosis. Furthermore, another role of *p21* is that it can be used as a predictive biomarker of response to therapy in TP53 and KRAS mutated NSCLC [44,45,46]. *NrCAM* is upregulated in a series of carcinomas like papillary thyroid carcinomas [47,48] and significant overexpression of the *NrCAM* in SCLC was also noted in comparison to normal lungs [49]. Of note, stable expression of the *NrCAM* ectodomain in NIH3T3 cells induced tumorigenesis in mice [50]. *RHOH* on the other hand, is a negative regulator of cell growth and survival. Rho GTPases regulate cell migration, proliferation, survival, and death. All these cellular processes are crucial for the maintenance of normal tissues, but also contribute to cancer progression [51]. For example, *RHOH* expression levels correlate with prostate cancer progression [52].

In H1975 cells silencing XIST lead to a highly significant down-regulation of 113 genes. One of these genes was *MYC*, a family of regulator genes and proto-oncogenes that code for transcription factors. It is well known that c-myc oncogene is frequently amplified in lung tumours and has been linked to their malignancy [53]. In a conditional model for the metastasis of NSCLC, c-myc played a crucial role in this process [54], and is overexpressed in 40–75% of NSCLC cases [55]. Inhibiting *MYC* on NSCLC in mice, lead to rapid regression of tumours with mild reversible side effects [56]. It is possible therefore that *MYC* can be targeted via silencing XIST. In contrast to A549 cells, *RHOH* was upregulated in H1975 cells following downregulation of XIST. This warrants further research as it might be a case of a differential expression in a cell- or sex-specific manner. Future studies should involve further research into the role of XIST using a wider repertoire of in vitro models. Our current cell lines have an EGFR mutant phenotype harbouring a T790M resistance mutation and a L858R sensitizing mutation (H1975), whereas the A549 cell line is KRAS mutant. Of note, mutations of the inactive X chromosome constitute a feature of tumorigenesis [57].

Gene enrichment analyses revealed that for A549 cells the main biological processes (in terms of percentage of genes involved) are signal transduction, cell communication, energy pathways, and metabolism; with the latter two having a number of same genes involved. For example, *CYP24A1* has oncogenic properties in lung adenocarcinoma [58,59] and *AKR1B10* has been shown to induce the metastatic potential of lung cancer cells to the brain in vitro. MicroRNA-1304 inhibited the growth of an NSCLC cell line by targeting HMOX1 [60]. Similarly, enrichment analysis in siRNA-treated H1975 cells, revealed that a number of processes including cell communication and signal transduction. These involve genes like *MUC16* (CA125) whose levels relate to different stages of NSCLC [61], and *PTK7* that is associated with lymph node metastasis as well as ALK and EGFR mutations in lung cancer [62]. In addition, PTN another gene effected by XIST is a heparin-binding growth factor that is involved with tumour progression [63]. In the same cell line, XIST affected the expression of 13 different zinc finger proteins. This is of increasing importance since they constitute the largest transcription factor family in the human genome and have been implicated in cancer progression [64].

Collectively, our study raises issues regarding detection of lncRNA using RNA-seq and provides a novel insight into a sex-specific role of XIST that warrants further investigation that can be of translational value in terms of the development of biomarkers and new therapeutic approaches targeting XIST and/or components of the signaling pathways it regulates in NSCLC.

## 4. Materials and Methods

### 4.1. Cell Lines

Two cell lines were used as in vitro models for lung cancer, A549 (ATCC^®^ CCL-185™, Gaithersburg, MD, USA) and H1975 (ATCC^®^ CRL-5908™, Gaithersburg, MD, USA). A549 cells were grown in complete DMEM (Dulbecco’s Modified Eagle’s Medium, Gibco, Fisher Scientific, Loughborough, UK) with 10% fetal bovine serum (FBS, Gibco, Fisher Scientific, Loughborough, UK), 1% penicillin/streptomycin (Gibco, Fisher Scientific, Loughborough, UK) and 1% L-Glutamine (Gibco, Fisher Scientific, Loughborough, UK). H1975 cells were grown in RPMI + 1% L-Glutamine (Gibco, Fisher Scientific, Loughborough, UK), 10% FBS and 1% Pen/strep. Cell lines were cultured at 37°C, in a 5% CO_2_ environment. 

### 4.2. siRNA for XIST

We used siRNA targeted to XIST (SMARTpool: ON-TARGETplus, Dharmacon, CO, USA) to suppress XIST gene expression in A549 and H1975 cells. Both cell lines were seeded at 2 × 10^4^ per well in 6-well plates. The siRNA transfection was carried out once at a concentration of 25 nmol/L per well/dish using Dharmafect 1 transfection reagent and serum-free media, a scrambled control was also included (ON-TARGETplus Non-targeting Pool; Horizon Discovery, UK). Cells were cultured in 3 biological replicates for 48 and 72 h. Then, 24 h post-transfection the medium was changed and replaced with fresh antibiotic-free medium [31].

### 4.3. RNA Isolation, cDNA Synthesis, and qPCR

RNA was extracted from cell lines, using the GenElute mRNA MiniPrep Kit (Merck, Feltham, UK), a silica membrane/spin column method, and stored at −80 °C until further use. cDNA was synthesized from mRNA using Superscript II (Fisher Scientific, Loughborough, UK). cDNA concentration was normalized using RNA concentrations determined by NanoDrop (Fisher Scientific, Loughborough, UK) and was synthesized to a concentration of 1000 ng according to manufacturer’s instructions.

### 4.4. RT-qPCR

Relative expression of the genes of interest was assessed by quantitative PCR using the SYBR green PCR master mix ROX (ABI, Fisher Scientific, Loughborough, UK) (Table 1). 18S RNA was used as the endogenous control and the targets investigated were manufactured by Sigma-Aldrich. Then, 1 μL from the cDNA (1000 ng/mL) and 0.5 μL of the primers (10 μM) were used for each reaction. All reactions were run in triplicate and a non-template control (NTC) was included in every run.

### 4.5. RNAseq

RNA sequencing was carried out on lung cancer cell lines treated with siRNA XIST for 48 h. All samples were sequenced at 75bp paired-end according to Illumina specifications. The data were analyzed using open-source software from the Tuxedo suite. Reads were mapped to the human reference genome hg19 using the GENCODE 24 annotations [6], with TopHat2 [23] (Bowtie 2) under standard conditions. The alignments were filtered for high-quality hits with a minimum selection threshold score of 30. The mapped reads were assembled into transcripts and quantified using the Cufflinks package [34]. Functional enrichment analyses and Venn diagrams were performed in the open software FunRich. The statistical cut-off of functional enrichment analyses using this stand-alone software was kept at default setting with a *p*-value < 0.05 after Bonferroni correction.

### 4.6. Bioinformatic Analysis

**Figure 8:**
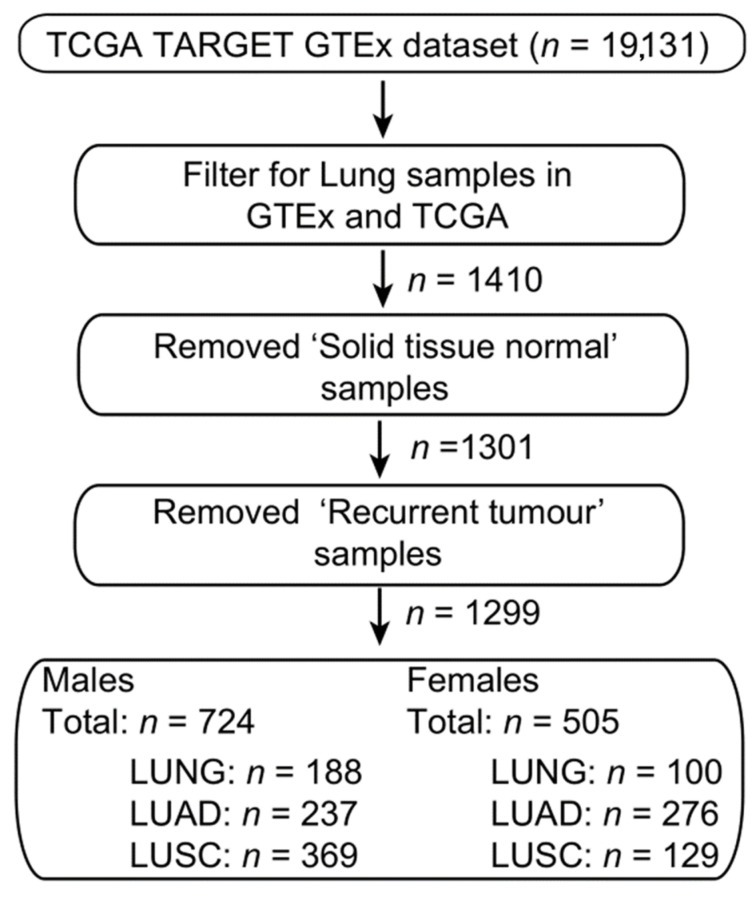
Workflow for the extraction and pre-processing of TCGA and GTEX samples from the UCSC Xenabrowser.

The XIST expression data and associated sample survival information were extracted from the UCSC Xenabrowser (xenabrowser.net), cohort “TCGA TARGET GTEx”. Samples from the publicly available datasets from The Cancer Genome Atlas project (TCGA), and Genotype-Tissue Expression project (GTEx) labelled as “primary tumour” and respectively “normal tissue” were selected for further processing (Figure 8). All expression data were cross-study normalized by UCSC using TOIL [35]. All expression data is presented in units of log2(norm_count + 1). Disease-specific phenotype data related to cancer stage, patient age, gender, and smoking status were extracted from the UCSC Xenabrowser TCGA Lung Adenocarcinoma (LUAD) and TCGA Lung Squamous Cell Carcinoma (LUSC) cohorts.

The Wilcoxon test was used to compare the gene expression level between two cohorts and Spearman’s rank test was used to determine the correlation coefficient between two gene expression patterns. All statistical tests performed in R v3.5.0. A summary of the data pre-processing steps is shown in Figure 2. T-SNE analysis was performed using the RtSNE v0.15 package in R v3.5.0. (www.rstudio.com/)

## 5. Conclusions

Our study provides a novel insight into the role of XIST in lung cancer. Apart from exhibiting sexual dimorphism in terms of expression, the downregulation of this lncRNA in NSCLC appears to influence differential signalling cascades and genes in male versus female cancer cell lines. The co-expression of *XIST*, *TSIX*, *hnRNPu*, *Bcl-2*, and *BRCA1* provided a strong collective feature to discriminate between controls and LC, implying a diagnostic potential.

## Figures and Tables

**Table 1:** Primers for targets selected.

Primer	Sequence 5′–3′
18S RNA-Forward	ATGGCCGTTCTGAGTTGGTG
18S RNA-Reverse	CGCTGAGCCAGTCAGTGTAG
PRKCQ-Forward	CTTGTGGCAGCTTTGGATGT
PRKCQ-Reverse	CGTTTCTGACGCACATGTTT
NrCAM-Forward	TTGTGCAAAGAGGGAGCATG
NrCAM-Reverse	GGGCAGTTCCCTGTTGTCCT
CDKN1A-Forward	GCAGACCAGCATGACAGATTT
CDKN1A-Reverse	GGATTAGGGCTTCCTCTTGGA
RHOH Forward	GAGAAGTAACATTCTGCAAATCGC
RHOH Reverse	AGCACACGCCATTCAGCAAG
XIST-Forward	AGGTCAGGCAGAGGAAGTCA
XIST-Reverse	AGGTCAGGCAGAGGAAGTCA
ROX1-AS1 Forward	CTAGTTAGCAGGGGCAGCAC
PROX1-AS1 Reverse	AACAGAGAGGCGTGGAAGAA
MYC-Forward	TGGACATCCGCAAAGACCTGTAC
MYC-Reverse	TCAGGAGGAGCAATGATCTTGA
SFTA1P-Forward	CAGCATTCCAGGTGGGCTTT
SFTA1P-Reverse	CCTTGTTTGGCTTACTCGTGC

qPCR data of relative gene expression was analyzed using the ΔΔCt method whereby the endogenous control Ct is subtracted from the gene of interest Ct to calculate ΔCt, and a relative quantity value was calculated by finding 2^−^^ΔΔCt^. A Student’s unpaired *t*-test was used to calculate statistical significance (*p* < 0.05).

## Supplementary Materials

The following are available online at https://www.mdpi.com/2072-6694/12/12/3499/s1, Figure S1: Heatmap of gene expression of *XIST*, *TSIX*, *HNRNPU*, *BCL-2* and *BRCA1* for each individual sample collected from TCGA and GTEx. Sexes and tissue types (healthy lung, lung adenocarcinoma/LUAD and lung squamous cell carcinoma/LUSC) are labelled as two additional columns. Gene expressions have units of log2(norm_count + 1), normalized by upper quartile method. Individual sample IDs were labeled on the left side of the heatmap. Total number of samples is 1299, data directly obtained from Xenabrowser cohort, “TCGA TARGET GTEx”, Figure S2: tSNE clustering of various gene combinations differentiating for gender and sample type: (a) XIST/TSIX, (b) XIST/HNRNPU, (c) XIST/BCL2, (d) XIST/BRCA1, (e) XIST/TSIX/HNRNPU, (f) XIST/HNRNPU/BCL2, (g) XIST/TSIX/BRCA1, (h) XIST/TSIX/BCL2, (i) XIST/BCL2/BRCA1, (j) XIST/HNRNPU/BRCA1, (k) XIST/TSIX/HNRNPU/BRCA1, (l) XIST/BCL2/BRCA1/HNRNPU, (m) XIST/TSIX/BCL2/BRCA1, (n) XIST/TSIX/BCL2/HNRNPU. Figure S3: Correlation with regression line for gene expression (in units if log2(norm_count + 1)) for all pairwise combinations of genes from XIST, TSIX, hnRNPu, TSIX, Bcl-2 and BRCA1. Spearman’s rank correlation test was used to generate the correlation coefficient represented as R in the graph, p represents *p*-value. Healthy lung, LUAD, and LUSC were compared separately in both sexes. Total sample number is 1299, 794 derived from male patients and 505 from female patients. There are 116 samples from male patients with 0 expression in XIST, 461 in TSIX, 1 in hnRNPu, Bcl-2, and BRCA1. In female patients derived samples, 17 show zero expression for TSIX gene. All samples with zero expression in both genes investigated were removed the analysis. Table S1: Spreadsheet with the differentially expressed genes in A549 and H1975 cell lines following siRNA of XIST.
