# Supplementary material for: In Silico and In Vitro Analysis of lncRNA XIST Reveals a Panel of Possible Lung Cancer Regulators and a Five-Gene Diagnostic Signature
Source: Cancers (Basel). 2020 Nov 24;12(12):3499. doi: 10.3390/cancers12123499 (PMC7760781; doi:10.3390/cancers12123499)
Supplement: Supplementary file 1 [file cancers-12-03499-s001.zip › supplementary Figure S1-S3.docx]

**Supplementary Materials:**

In Silico and In Vitro Analysis of lncRNA XIST Reveals a Panel of Possible Lung Cancer Regulators and a Five-Gene Diagnostic Signature

Periklis Katopodis Qiduo Dong, Heerni Halai, Cristian I. Fratila, Andreas Polychronis,
Vladimir Anikin, Cristina Sisu and Emmanouil Karteris


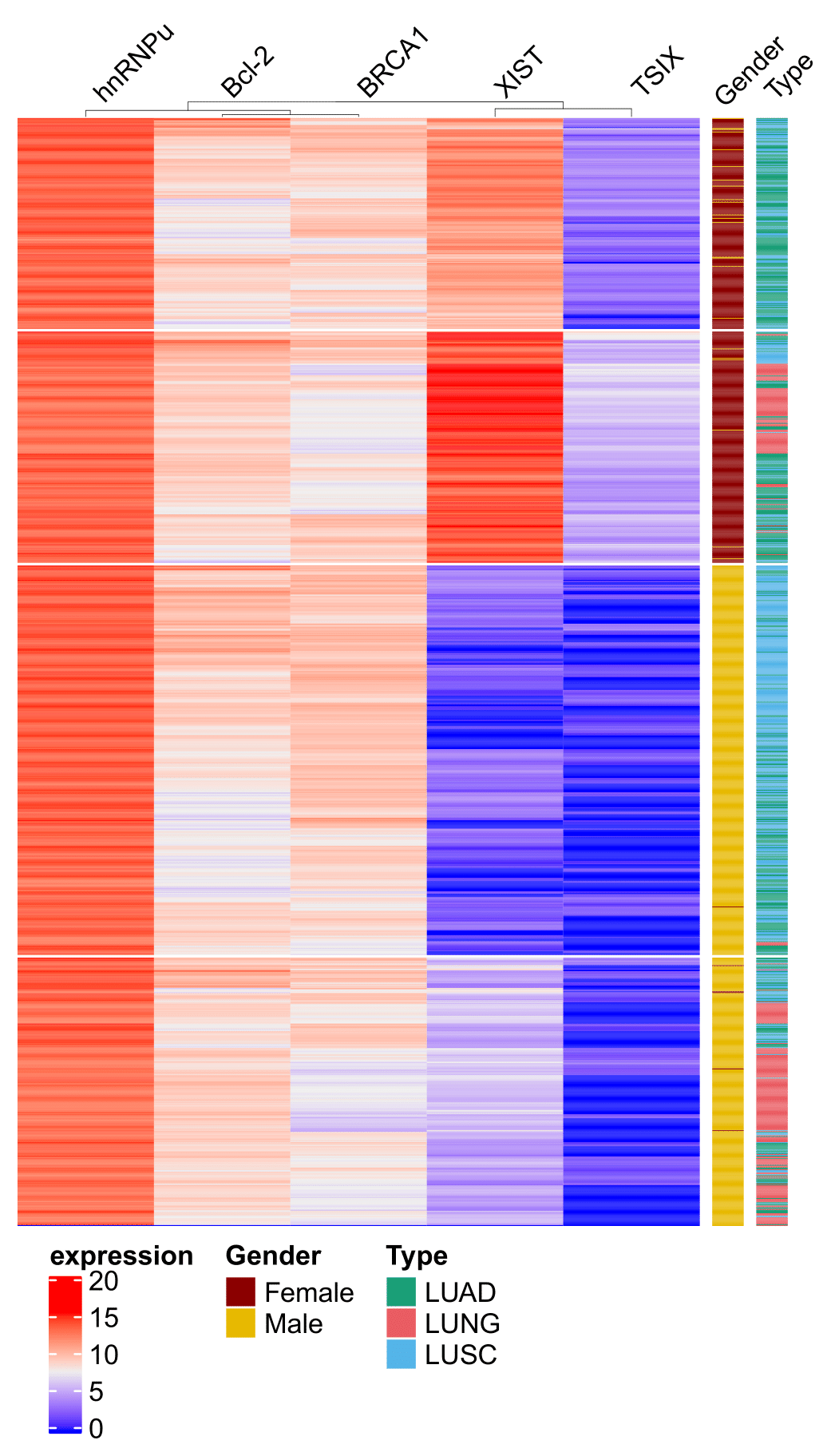


**Figure S1.** Heatmap of gene expression of *XIST*, *TSIX*, *HNRNPU*, *BCL-2* and *BRCA1* for each individual sample collected from TCGA and GTEx. Sexes and tissue types (healthy lung, lung adenocarcinoma/LUAD and lung squamous cell carcinoma/LUSC) are labelled as two additional columns. Gene expressions have units of log2(norm_count + 1), normalized by upper quartile method. Individual sample IDs were labeled on the left side of the heatmap. Total number of samples is 1299, data directly obtained from Xenabrowser cohort, “TCGA TARGET GTEx”,


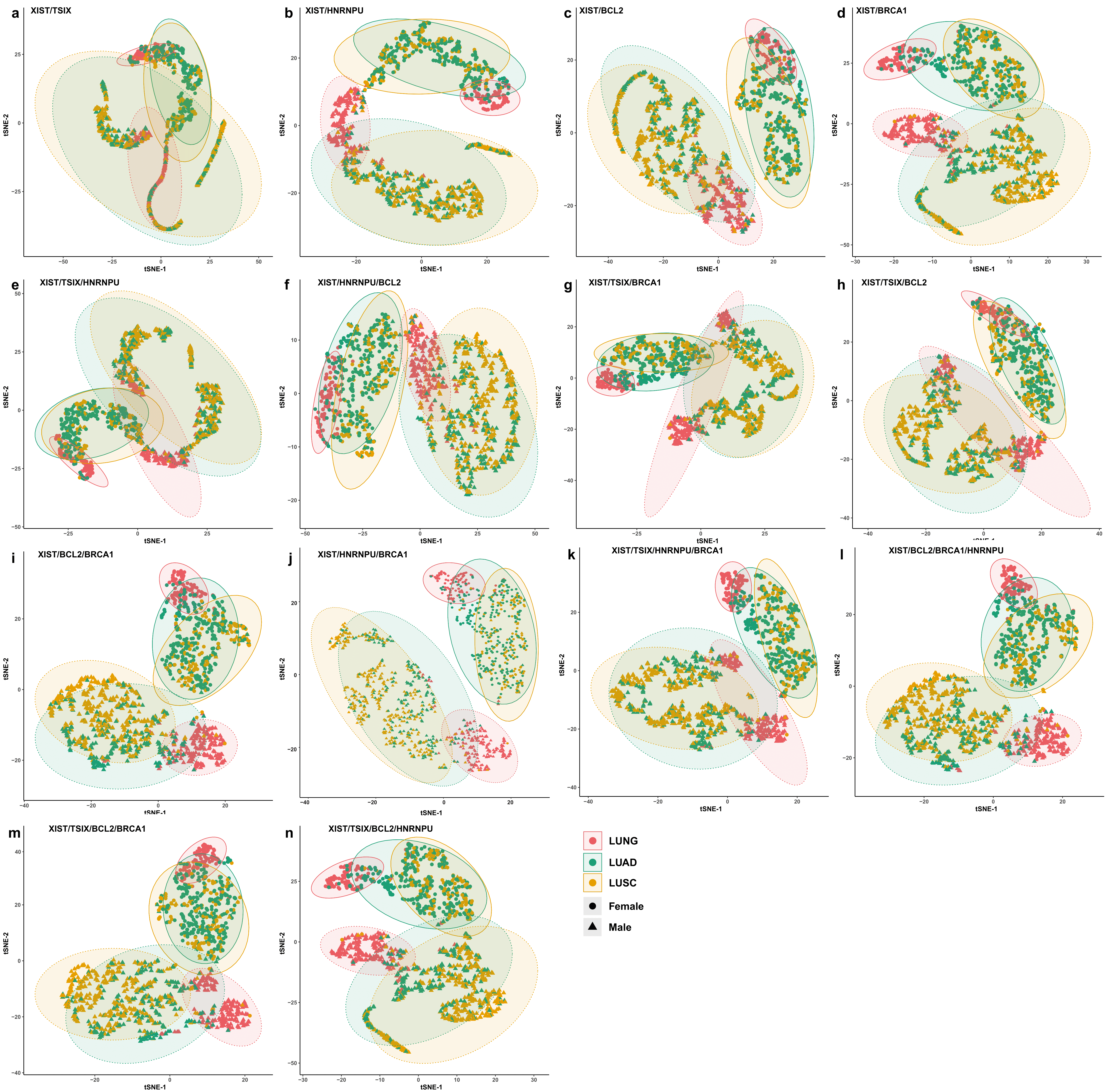


**Figure S2.** tSNE clustering of various gene combinations differentiating for gender and sample type: (a) XIST/TSIX, (b) XIST/HNRNPU, (c)XIST/BCL2, (d) XIST/BRCA1, (e) XIST/TSIX/HNRNPU, (f) XIST/HNRNPU/BCL2, (g) XIST/TSIX/BRCA1, (h)XIST/TSIX/BCL2, (i) XIST/BCL2/BRCA1, (j) XIST/HNRNPU/BRCA1, (k) XIST/TSIX/HNRNPU/BRCA1, (l) XIST/BCL2/BRCA1/HNRNPU, (m) XIST/TSIX/BCL2/BRCA1, (n) XIST/TSIX/BCL2/HNRNPU.


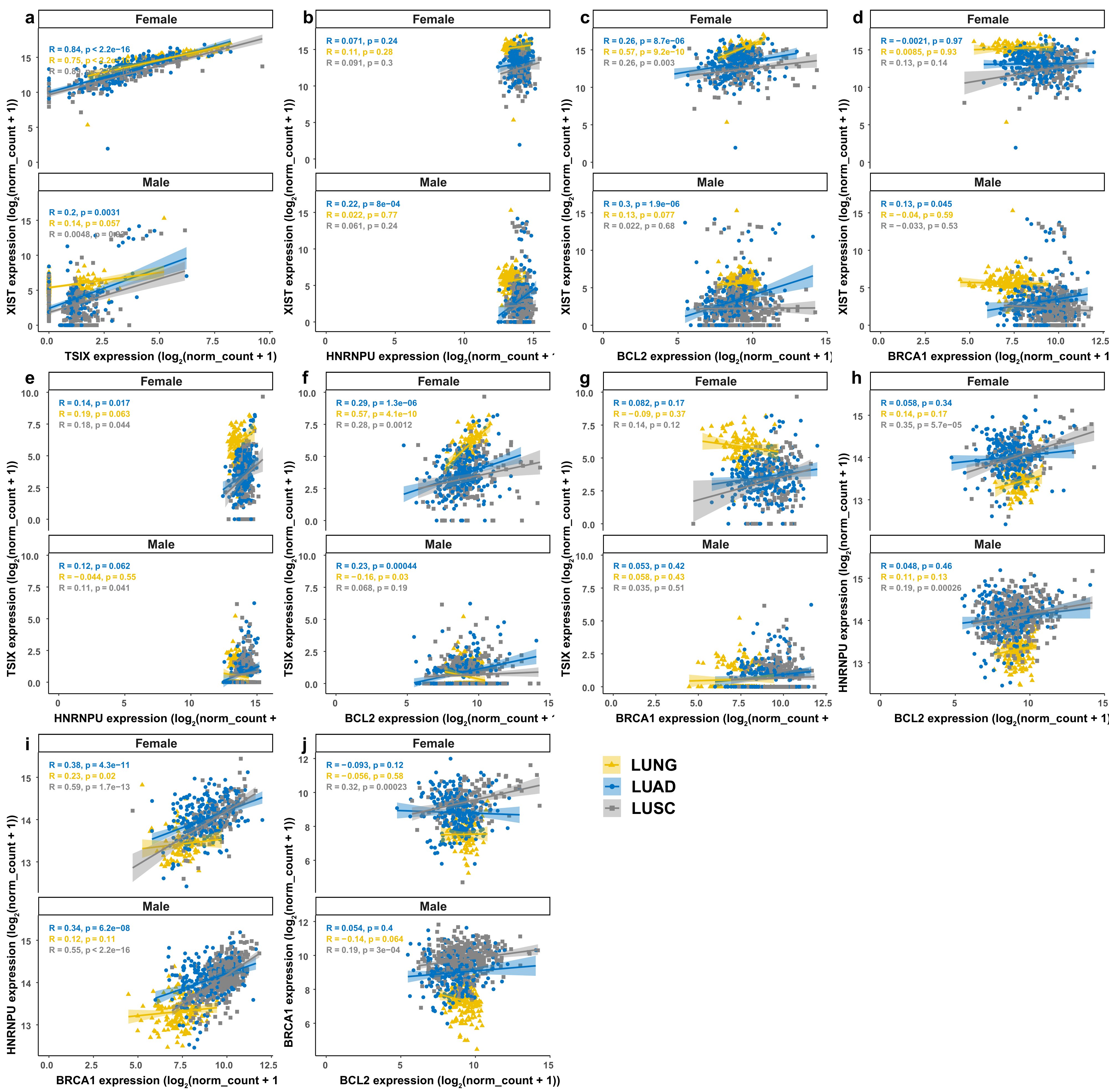


**Figure S3.** Correlation with regression line for gene expression (in units if log2(norm_count + 1)) for all pairwise combinations of genes from XIST, TSIX, hnRNPu, TSIX, Bcl-2 and BRCA1. Spearman’s rank correlation test was used to generate the correlation coefficient represented as R in the graph, p represents *p*-value. Healthy lung, LUAD, and LUSC were compared separately in both sexes. Total sample number is 1299, 794 derived from male patients and 505 from female patients. There are 116 samples from male patients with 0 expression in XIST, 461 in TSIX, 1 in hnRNPu, Bcl-2, and BRCA1. In female patients derived samples, 17 show zero expression for TSIX gene. All samples with zero expression in both genes investigated were removed the analysis.
